# A Regression Analysis on Steam Gasification of Polyvinyl Chloride Waste for an Efficient and Environmentally Sustainable Process

**DOI:** 10.3390/polym15132767

**Published:** 2023-06-21

**Authors:** Rezgar Hasanzadeh, Rzgar M. Abdalrahman

**Affiliations:** 1 Department of Mechanical Engineering, Faculty of Engineering, Urmia University, Urmia 5756151818, Iran; 2 Department of Mechanical Engineering/Production, College of Engineering, Sulaimani Polytechnic University, Sulaimani 70-236, Kurdistan, Iraq

**Keywords:** machine learning, polyvinyl chloride, plastic gasification, regression model, environmental sustainability

## Abstract

Over the last few years, researchers have shown a growing interest in polyvinyl chloride (PVC) gasification and have conducted several studies to evaluate and enhance the process. These studies have recognized that processing parameters have a crucial impact on the assessment of PVC gasification. Despite this, there has been limited exploration of the use of machine learning techniques, particularly regression models, to optimize PVC waste gasification. This study aims to investigate the effectiveness of regression models as machine learning algorithms in predicting the performance of PVC waste gasification. The study uses data collected through a validated thermodynamic model, and three different regression models are tested and compared in detail. Cold gas efficiency and normalized carbon dioxide emission are predicted using linear, quadratic, and quadratic with interaction algorithms. The outcomes for emission algorithms reveal that the linear emission algorithm possesses a high R-square value of 97.49%, which indicates its strong predictive capability. Nevertheless, the quadratic algorithm outperforms it, exhibiting an R-square value of 99.81%. The quadratic algorithm with an interaction term, however, proves to be the best among them all, displaying a perfect R-square value of 99.90%. A similar observation is detected for the cold gas efficiency algorithms. These findings suggest that the quadratic algorithm with an interaction term is superior and has a greater predictive accuracy. This research is expected to provide valuable insight into how regression algorithms can be used to maximize the efficiency of PVC waste gasification and reduce its associated environmental concerns.

## 1. Introduction

The versatility, durability, and cost-effectiveness of plastic have made it an essential material in various industries, from packaging to construction [1]. Despite the many benefits of plastics, their overuse and improper disposal have led to a severe environmental crisis [2]. Plastic waste has become a significant threat to the environment, with millions of tons of plastic ending up in oceans and landfills every year [3]. The problem with plastic waste is that it takes hundreds of years to decompose, and it often ends up in landfills or oceans, causing harm to wildlife and the environment [4]. This has led to a massive accumulation of plastic waste in our landfills, oceans, and water bodies [5].

Plastic waste has various environmental impacts that are detrimental to our planet. It harms wildlife, disrupts ecosystems, and affects human health [6]. The accumulation of plastic waste in our oceans also leads to the formation of microplastics, which are small plastic particles that are harmful to marine animals and have the potential to enter the food chain [7]. The problem of plastic waste also affects human health, as it leaches toxic chemicals into the soil and water, which can lead to health problems such as cancer, birth defects, and hormonal imbalances [8]. The burning of plastic waste also releases toxic gases into the atmosphere, leading to air pollution and respiratory problems [9].

It is critical to identify effective ways to treat and manage plastic waste [10]. There are various methods of treating and managing plastic waste, and thermochemical techniques are among the most effective [11]. Thermochemical techniques are a group of methods that use heat to break down plastic waste into its constituent parts. This process involves the application of high temperatures and pressure to plastic waste to break down the complex molecules into simpler elements [12]. The resulting products can be further processed to create useful materials. The most important thermochemical techniques include incineration, pyrolysis and gasification [13].

Plastic waste incineration, while considered a common method of waste disposal, has numerous disadvantages that can lead to negative impacts on both the environment and human health [14,15]. One of the major disadvantages of plastic waste incineration is the release of harmful pollutants, such as dioxins and furans, into the air, which can cause respiratory issues and even cancer. Incineration can also lead to the production of toxic ash, which must be carefully disposed of to prevent further harm to the environment [16]. With these disadvantages in mind, it is crucial to consider alternative methods of waste disposal that prioritize sustainability and environmental protection.

Plastic waste pyrolysis is a process that converts plastic waste into fuel oil, carbon black, and gases through high-temperature decomposition [17]. While this process has been touted as an eco-friendly solution to the plastic waste problem, it is not without its disadvantages [18]. One major disadvantage of plastic waste pyrolysis is the emission of harmful gases such as carbon dioxide, carbon monoxide, and volatile organic compounds [18,19]. Another disadvantage of plastic waste pyrolysis is the quality of the fuel produced. The fuel oil produced from the process is of low quality and cannot be used in most combustion engines [20]. Overall, while plastic waste pyrolysis may seem like a good solution to the plastic waste problem, it is important to consider its disadvantages and explore alternative solutions.

Gasification is another thermochemical technique that involves heating plastic waste in the presence of oxygen or steam, and is the process of converting plastic waste into a synthetic gas, which can be used to generate electricity or fuel [21]. Gasification of plastic waste has several advantages. Firstly, it reduces the amount of plastic waste that ends up in landfills [22]. Secondly, gasification of plastic waste can help in the generation of clean energy. By using clean energy generated from plastic waste gasification, we can reduce our carbon footprint and contribute to a cleaner environment [23]. Thirdly, gasification of plastic waste can help in the reduction of air pollution. This can help in improving the air quality and reducing the impact of air pollution on public health [24].

Polyvinyl chloride, commonly known as PVC, is a versatile and widely used thermoplastic polymer that has a variety of applications across numerous industries [25]. PVC, which has two forms, hard and soft PVC, is made by polymerizing vinyl chloride monomers, and it is a popular material due to its low cost, durability, and ease of processing. PVC can be easily molded, extruded, and fabricated into a wide range of products, making it a preferred choice for manufacturers [26].

PVC has a wide range of applications, including construction, automotive, healthcare, electrical, and packaging [27]. One of the biggest advantages of PVC is its low cost, making it a cost-effective choice for manufacturers. PVC is also durable and long-lasting, making it a preferred material for products that require longevity. It is lightweight, making it easy to transport, and it is also easy to install and maintain. PVC is also resistant to chemicals, making it a suitable material for products that require resistance to corrosive substances [28]. Due to its wide application range, it is predictable that PVC waste will grow rapidly, and it is necessary to provide suitable treatment methods and gasification process plays a vital role in this manner [29].

PVC is a versatile and widely used thermoplastic polymer that has numerous applications across various industries. Its low cost, durability, and ease of processing make it a preferred choice for manufacturers. Its wide application range results in huge amount of waste generation, raising serious environmental and health concerns. PVC waste generation is a significant environmental challenge that requires a collective effort to overcome. To combat the problem of PVC waste and its environmental impacts, it is crucial to adopt sustainable practices such as reducing, reusing, and recycling PVC waste. Thermochemical techniques, such as gasification, are among the most effective methods of treating PVC waste. Gasification can break down PVC waste into useful products that can be used to create fuels, chemicals, or different forms of energy. PVC gasification not only helps in reducing the amount of PVC waste that ends up in landfills but also generates clean energy and reduces air pollution. With the increasing need for sustainable solutions, PVC waste gasification can play a crucial role in addressing the environmental challenges we face today.

In recent years, the increasing interest in PVC gasification has resulted in numerous studies aimed at evaluating and improving this process. However, these studies have acknowledged that processing parameters play a critical role in assessing the PVC gasification process. Despite this, there has been limited research on the use of machine learning techniques to optimize PVC waste gasification, particularly in the application of regression models. This study aims to determine the effectiveness of regression models as machine learning algorithms in predicting the performance of PVC waste gasification. Data for this study were collected through a validated thermodynamic model, and three different regression models were tested and compared in detail. The results of this study emphasize the significance of employing regression models as machine learning algorithms in the field of PVC waste gasification.

## 2. Machine Learning Algorithm

Machine learning is a field of study that focuses on developing algorithms and statistical models that enable computer systems to improve their performance on specific tasks without being explicitly programmed [30]. This innovative technology has been transforming the way businesses operate and has the potential to revolutionize various industries. Machine learning algorithms can analyze large amounts of data and make predictions or decisions based on that information [31]. They can identify patterns and relationships that humans may not be able to detect, providing insights that can be used to optimize business operations. Machine learning has numerous applications in a wide range of industries, from healthcare to finance and manufacturing [32]. The benefits of machine learning are numerous. By automating complex decision-making processes, machine learning algorithms can save businesses time and money [33]. They can also improve the accuracy of predictions, leading to better decision-making and improved outcomes. Furthermore, machine learning algorithms can be trained to adapt to changing conditions, allowing businesses to stay ahead of the curve and respond to new challenges as they arise [34]. However, machine learning is not without its challenges. One major obstacle is the need for large amounts of high-quality data to train the algorithms. Additionally, machine learning algorithms can be susceptible to bias if the data used to train them are not representative of the real-world population [35]. To address these challenges, it is important to have a thorough understanding of machine learning algorithms and their limitations.

Applications of machine learning in the field of gasification are attracting considerable attention. Khan et al. [36] utilized machine learning for prediction of hydrogen production in gasification of sewage sludge using supercritical water. Their findings revealed that ensembled learning tree technique had the best performance in prediction of hydrogen yield. Li et al. [37] used different machine learning techniques for prediction of biomass waste gasification. They found that gradient boosting regression had the best performance in predicting the syngas composition. Hasanzadeh et al. [32] utilized the response surface methodology as a machine learning tool for predicting the behavior of polyethylene waste gasification. They showed that this technique can predict the performance of polyethylene waste gasification with low root mean squares, indicating high accuracy. Yang et al. [38] used machine learning algorithms to predict the gasification process of municipal solid waste. They showed that gradient boost regressor had high accuracy when estimating waste gasification. Although the applications of machine learning techniques in the field of gasification are growing quickly, no studies were found that addressed this topic with regard to PVC gasification.

One of the most popular machine learning algorithm types is the regression model. It is a statistical technique that is used to estimate the relationship between a dependent variable and one or more independent variables [39]. Regression models are widely used in various industries.

Regression models are primarily used for prediction and forecasting purposes. They help in predicting the future outcomes based on historical data. This is done by estimating the relationship between the dependent and independent variables. Regression models come in various forms, including linear regression, logistic regression, and polynomial regression [40]. Each regression model is used to analyze different types of data and solve different types of problems.

Linear regression is one of the most popular regression models used in machine learning. It is a simple technique that is used to estimate the relationship between two variables. The output of the linear regression model is a continuous value that can be used to predict the value of the dependent variable [41]. Polynomial regression is used when the relationship between the dependent and independent variables is nonlinear. It is used to estimate the relationship between the variables by fitting a polynomial curve to the data [42]. Regression models have several advantages over other machine learning algorithms. They are easy to understand and interpret and require less computation power.

The application of machine learning in the gasification process has the potential to improve the efficiency and reduce the cost of the process. By using machine learning techniques such as regression models, operators can optimize the process parameters and improve the gasification efficiency. Real-time monitoring using machine learning algorithms can also help operators to detect anomalies and prevent downtime, further improving the efficiency of the process.

Therefore, regression models are employed for predicting the gasification performance of the PVC waste in this study. Three different regression models are considered, and their performances are compared. In the first type, only the linear terms of the independent variables ($X_{i}$) are considered in the model. This model is named the linear regression model. In the second type, the quadratic terms of the independent variables ($X_{i}^{2}$) are also considered and this model is called the quadratic model. In the third type, the interaction terms between the independent variables ($X_{i}X_{j}$) are also considered and this model is named the quadratic model considering interactions.

The first type is a linear regression model, as follows [43]:

(1) $$R=\alpha +\overset{n}{\underset{i=1}{\sum }}\beta_{i}X_{i}$$

where R indicates the response output to be predicted based on the independent variables ($X_{i}$), α denotes the constant values and $\beta_{i}$ refers to the linear coefficients.

The second type is a quadratic regression model, as follows [43]:

(2) $$R=\alpha +\overset{n}{\underset{i=1}{\sum }}\beta_{i}X_{i}+\overset{n}{\underset{i=1}{\sum }}\gamma_{i}X_{i}^{2}$$

where $\gamma_{i}$ indicates the square coefficients.

The third type is a quadratic regression model considering the interaction terms, as follows [44]:

(3) $$R=\alpha +\overset{n}{\underset{i=1}{\sum }}\beta_{i}X_{i}+\overset{n}{\underset{i=1}{\sum }}\gamma_{i}X_{i}^{2}+\overset{n}{\underset{i=1}{\sum }}\overset{n}{\underset{j=1,i\neq j}{\sum }}\delta_{ij}X_{i}X_{j}$$

where $\delta_{ij}$ refers to the interaction coefficients.

## 3. Gasification of PVC Waste

The gasification process with steam agents is a highly efficient method for converting solid fuels into gaseous fuels. The process involves the reaction of a solid fuel with steam at high temperatures, resulting in the production of a combustible gas mixture known as syngas [45]. The gasification process occurs in a gasifier, which is a reactor vessel that is designed to withstand high temperatures and pressures.

The gasification process with steam agents takes place in several distinct stages. Initially, the solid fuel is fed into the gasifier, where it is heated to a high temperature. At this temperature, the fuel undergoes thermal decomposition, releasing volatile gases and tars [46]. These gases and tars then react with the steam, resulting in the formation of syngas. The syngas produced in this stage typically contains carbon monoxide, hydrogen, and methane [47].

The composition of PVC waste is considered to be ${\mathrm{CH}}_{m_{1}}N_{m_{2}}O_{m_{3}}$, where $m_{i}$ refers to the molar ratio of *i*th element to the carbon and its steam gasification reactions is assumed as follows [48]:

(4) $$xH_{2}O+{\mathrm{CH}}_{m_{1}}N_{m_{2}}O_{m_{3}}+yH_{2}O\;\to \varepsilon_{1}\mathrm{CO}+\varepsilon_{2}H_{2}+\varepsilon_{3}{\mathrm{CH}}_{4}+\varepsilon_{4}H_{2}O+\varepsilon_{5}{\mathrm{CO}}_{2}$$

where x denotes the feed rate of steam agent and y indicates the moisture content of PVC waste.

The PVC waste considered in this study has a higher heating value of 18.04 MJ/kg with characteristics presented in Table 1. Since the weight percentage of nitrogen in the ultimate analysis is negligible, the production side of gasification reaction does not include any nitrogen element.

To determine the $\varepsilon_{i}$ values, a technique that combines Gibbs free energy with Lagrangian multipliers is utilized. This method takes into account both the total Gibbs free energy (G) and Lagrangian multipliers (λ), as follows [50]:

(5) $$G=\overset{n}{\underset{i=1}{\sum }}[\varepsilon_{i}\Delta {\bar{G}}_{i}+\varepsilon_{i}T\bar{R}\ln(\frac{\varepsilon_{i}}{\varepsilon_{t}})]$$

(6) $$\lambda =G+\overset{E}{\underset{r=1}{\sum }}[\lambda_{r}n-(\lambda_{r}\overset{C}{\underset{i=1}{\sum }}\varepsilon_{i}n_{i})]$$

The constraint of the elemental balance is as follows [50]:

(7) $$n=\overset{n}{\underset{i=1}{\sum }}\varepsilon_{i}n_{i}$$

where n refers to the total atom numbers and $n_{i}$ denotes the *i*th component atom number.

Therefore [51]:

(8) $$\Delta {\bar{G}}_{\mathrm{CO}}+T\bar{R}\ln(\frac{\varepsilon_{1}}{\varepsilon_{t}})+\lambda_{C}+\lambda_{O}=0$$

(9) $$\Delta {\bar{G}}_{H_{2}}+T\bar{R}\ln(\frac{\varepsilon_{2}}{\varepsilon_{t}})+2\lambda_{H}=0$$

(10) $$\Delta {\bar{G}}_{{\mathrm{CH}}_{4}}+T\bar{R}\ln(\frac{\varepsilon_{3}}{\varepsilon_{t}})+\lambda_{C}+4\lambda_{H}=0$$

(11) $$\Delta {\bar{G}}_{H_{2}O}+T\bar{R}\ln(\frac{\varepsilon_{4}}{\varepsilon_{t}})+2\lambda_{H}+\lambda_{O}=0$$

(12) $$\Delta {\bar{G}}_{{\mathrm{CO}}_{2}}+T\bar{R}\ln(\frac{\varepsilon_{5}}{\varepsilon_{t}})+\lambda_{C}+2\lambda_{O}=0$$

The five unknown values of $\varepsilon_{i}$ are calculated using the five equations mentioned above.

The energy balance is assumed to be as follows [52]:

(13) $${\bar{h}}_{f,\mathrm{PVC}}^{{}^{\circ}}+x\left({\bar{h}}_{f,H_{2}O_{l}}^{{}^{\circ}}+{\bar{h}}_{vap,H_{2}O}\right)+y\left({\bar{h}}_{f,H_{2}O}^{{}^{\circ}}+\Delta {\bar{h}}_{T,H_{2}O}^{{}^{\circ}}\right)+Q_{in}\;\;\;\;\;\;\;\;\;\;\;\;\;\;=\varepsilon_{1}\left({\bar{h}}_{f,\mathrm{CO}}^{{}^{\circ}}+\Delta {\bar{h}}_{T,\mathrm{CO}}^{{}^{\circ}}\right)+\varepsilon_{2}\left({\bar{h}}_{f,H_{2}}^{{}^{\circ}}+\Delta {\bar{h}}_{T,H_{2}}^{{}^{\circ}}\right)+\varepsilon_{3}\left({\bar{h}}_{f,{\mathrm{CH}}_{4}}^{{}^{\circ}}+\Delta {\bar{h}}_{T,{\mathrm{CH}}_{4}}^{{}^{\circ}}\right)+\varepsilon_{4}\left({\bar{h}}_{f,H_{2}O}^{{}^{\circ}}+\Delta {\bar{h}}_{T,H_{2}O}^{{}^{\circ}}\right)\;+\varepsilon_{5}\left({\bar{h}}_{f,{\mathrm{CO}}_{2}}^{{}^{\circ}}+\Delta {\bar{h}}_{T,{\mathrm{CO}}_{2}}^{{}^{\circ}}\right)$$

where [53]:

(14) $$\Delta {\bar{h}}_{T}=\int_{T_{0}}^{T}\left(\omega_{1}+\omega_{2}T+\omega_{3}T^{2}+\omega_{4}T^{3}\right)dT\;$$

The values of $\omega_{i}$ are as reported in [54].

Cold gas efficiency (CGE) of the PVC gasification is assumed to be as follows [55]:

(15) $$CGE=\frac{LHV}{E_{\mathrm{PVC}}+E_{\mathrm{steam}}+E_{Q}}$$

where LHV is the lower heating values of the syngas as follows:

(16) $$LHV=\varepsilon_{1}LHV_{\mathrm{CO}}+\varepsilon_{2}LHV_{H_{2}}+\varepsilon_{3}LHV_{{\mathrm{CH}}_{4}}$$

Normalized carbon dioxide emission (*Emission*) is assumed to be as follows [56]:

(17) $$Emission=\frac{m_{{\mathrm{CO}}_{2}}}{{\dot{\varepsilon }}_{1}HV_{\mathrm{CO}}+{\dot{\varepsilon }}_{2}LHV_{H_{2}}+{\dot{\varepsilon }}_{3}LHV_{{\mathrm{CH}}_{4}}}$$

where $m_{{\mathrm{CO}}_{2}}$ denotes the mass of carbon dioxide produced in the gasification.

**Figure 1:**
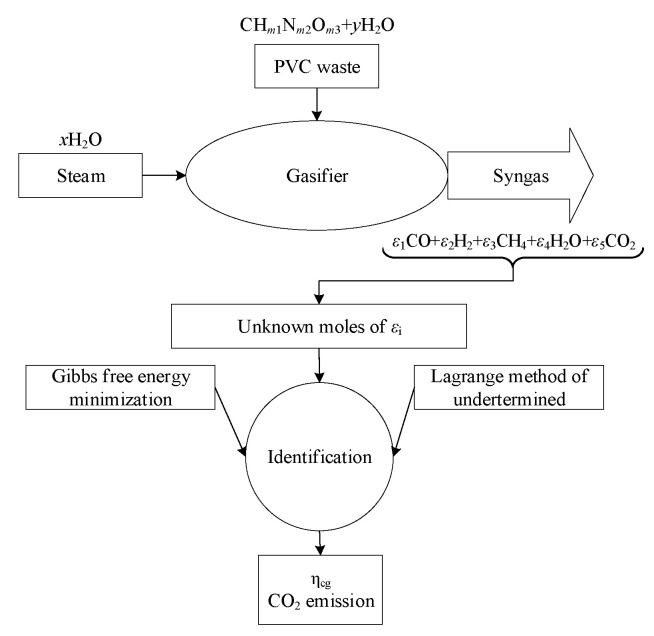
Modeling flowchart.

A flowchart of the modeling methodology is provided in Figure 1.

## 4. Results and Discussion

### 4.1. Validation of Gasification Modeling

Thermodynamic modeling is a widely used technique to simulate gasification processes. It involves the use of mathematical equations to predict the behavior of the gasification process under different conditions. However, the accuracy of the thermodynamic model largely depends on the data used to develop it. Validation of the thermodynamic model is necessary because gasification is a complex process that involves a variety of factors such as temperature, pressure, and composition of the feedstock. Therefore, validation of the thermodynamic model is crucial to ensuring its accuracy and reliability. Validation of the thermodynamic model involves comparing the model’s predictions with actual experimental data from the gasification process. This comparison helps to identify any discrepancies between the model’s predictions and actual experimental data. If discrepancies are found, the thermodynamic model can be adjusted to improve its accuracy.

**Figure 2:**
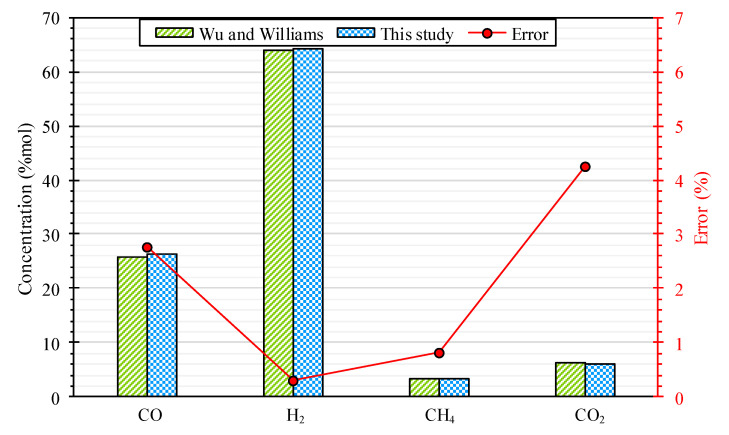
Validation of gasification modeling.

To ensure the steam plastic waste gasification model’s accuracy, the syngas composition of polypropylene gasification is compared with values reported by Wu and Williams [57] under similar conditions. They conducted an experimental gasification in a two-stage fixed-bed gasifier. The results are presented in Figure 2, indicating that the syngas composition predicted by this study’s modeling is consistent with [57] results. This suggests the accuracy and reliability of this study’s modeling. Moreover, the errors between the results are less than 5%, confirming the modeling’s validity. With the significant agreement between the syngas composition predicted by this modeling and the results presented in [57], it is concluded that the modeling is validated.

### 4.2. Process Evaluation

**Figure 3:**
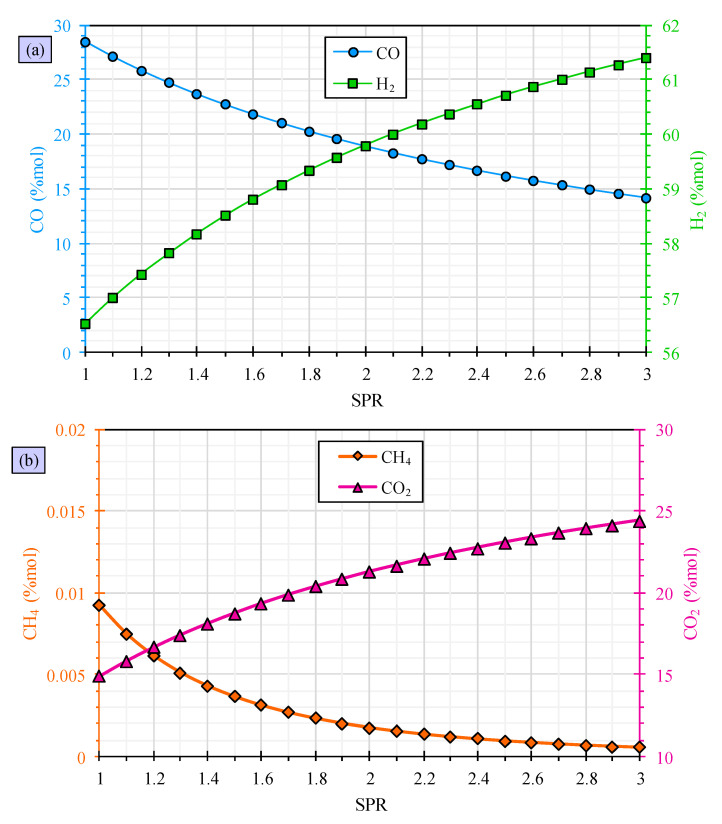
PVC waste gasification evaluation for carbon monoxide and hydrogen (**a**) and methane and carbon dioxide (**b**) versus steam-to-PVC waste ratio.

Figure 3 presents the findings of the assessment of the impact of steam-to-PVC waste ratio (SPR) on syngas composition. The results indicate that higher SPR leads to a reduction in carbon monoxide levels, an enhancement in hydrogen levels, a decrease in methane levels, and a rise in carbon dioxide levels. When the SPR is increased from 1 to 3, the amount of carbon monoxide decreases from 28.55% to 14.18%, while the hydrogen content increases from 56.53% to 61.40%. Additionally, the amount of carbon dioxide increases from 14.91% to 24.42%. While there is a slight decrease in methane content, it is not significant. The reasoning behind these observations can be supported by the water-shift reaction formula, which is presented as follows [58]:

(18) $$\mathrm{CO}+H_{2}O\to {\mathrm{CO}}_{2}+H_{2}$$

When the SPR is increased, the amount of $H_{2}O$ also increases, causing the reaction to shift towards the right side. As a result, more carbon monoxide is utilized and there is an increase in the production of carbon dioxide and hydrogen. These findings align with those of similar studies conducted on different feedstocks, as reported in literature such as that related to hazelnut shell gasification [59], biomass gasification [60], and wood residue gasification [61].

**Figure 4:**
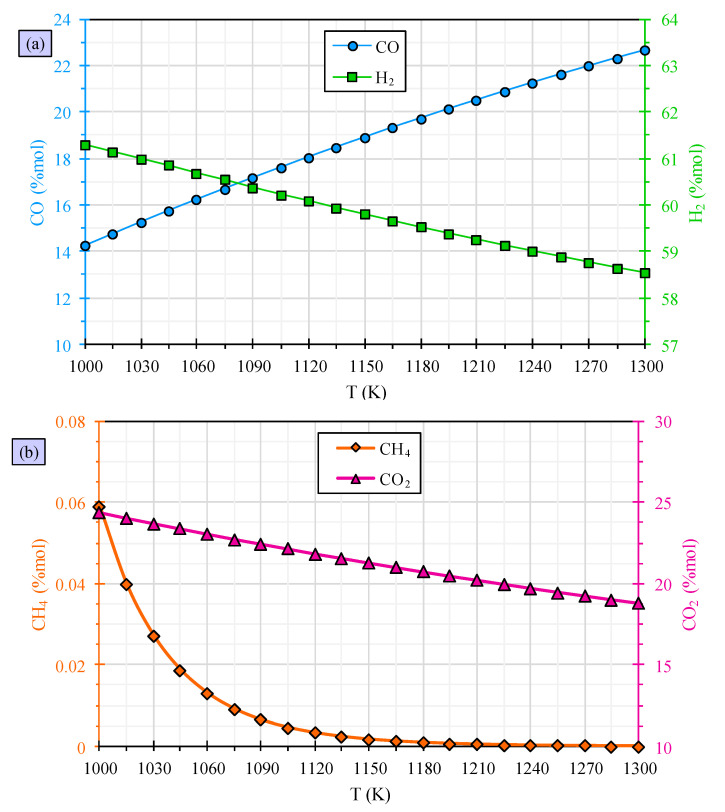
PVC waste gasification evaluation for carbon monoxide and hydrogen (**a**) and methane and carbon dioxide (**b**) versus temperature.

The influence of gasification temperature on the performance of PVC waste gasification is evaluated, and the results are presented in Figure 4. The evaluation shows that higher temperatures result in an increase in carbon monoxide content, a decrease in hydrogen content, a reduction in methane content, and a decline in carbon dioxide content. These results demonstrate the impact of temperature on the process of PVC waste gasification. Raising the temperature of gasification from 1000 to 1300 K results in an increase in carbon monoxide content from 14.24% to 22.65%, a decrease in hydrogen content from 61.30% to 58.54%, and a reduction in carbon dioxide content from 24.40% to 18.81%. The methane content is slightly reduced, but its impact is insignificant. The alterations made can be supported by the water–gas shift reaction, which is an exothermic chemical reaction. At elevated temperatures, the reaction shifts towards the left side, resulting in an increased consumption of hydrogen and carbon dioxide and a greater production of carbon monoxide. This phenomenon justifies the changes made.

The observations can also be explained by the Boudouard reaction, which is as follows, and this justification is also credible [62]:

(19) $$C+{\mathrm{CO}}_{2}\to 2\mathrm{CO}$$

This reaction is classified as endothermic, and has a tendency to shift towards the right side as the temperature rises. This results in an increased consumption of carbon dioxide and production of carbon monoxide.

These observations are consistent with the conclusions drawn in the literature that is currently available, such as sewage sludge gasification [63], municipal solid waste [64], and biomass stalk [60].

**Figure 5:**
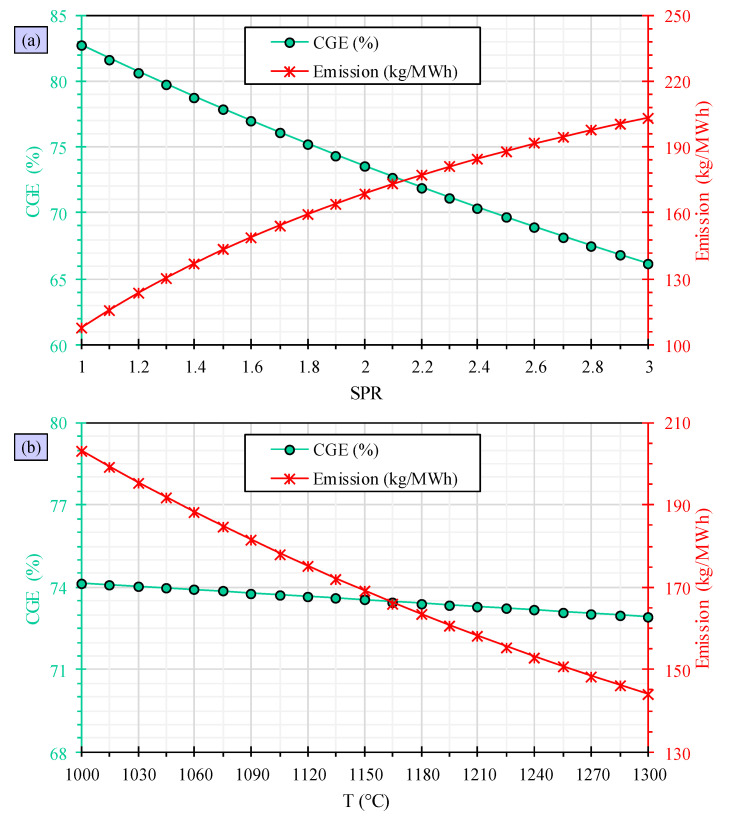
Evaluation of cold gas efficiency (CGE) and emission in PVC waste gasification versus steam-to-PVC waste ratio (**a**) and temperature (**b**).

The evaluation of the effects of SPR and temperature on the cold gas efficiency and emission of PVC waste gasification is conducted, and Figure 5 demonstrates the outcomes. Based on the findings, when the SPR is increased, it leads to a decline in cold gas efficiency and an increase in emission. The reduction in cold gas efficiency is almost 17% when the SPR is increased from 1 to 3, resulting in a decrease from 82.70% to 66.16%. Moreover, the emission increases from 108 to 203.6 kg/MWh, which is an increase of 89%. These results indicate that the increase in SPR has a negative impact in the performance of PVC waste gasification. The behaviors mentioned are reasonable and can be explained by the changes made to the syngas composition and the increased amount of steam fed into the reactor. However, it is important to note that reducing the carbon monoxide content and introducing more steam energy into the gasifier can have a negative impact on the efficiency of cold gas. Additionally, the higher levels of emissions observed can be attributed to the increase in carbon dioxide content at higher SPRs.

According to the findings, the cold gas efficiency remains largely unchanged, despite a temperature increase from 1000–1300 K. The cold gas efficiency only slightly decreases from 74.16% to 72.91%. This can be attributed to the fluctuations in carbon monoxide and hydrogen levels in syngas composition. The improvement in carbon monoxide content and the decline in hydrogen content cancel each other out, resulting in a constant cold gas efficiency. Based on these findings, there is a significant decrease in emission when the temperature is raised. Specifically, when the temperature is raised from 1000–1300 K, the emission decreases from 203.1 to 144.0 kg/kWh, which is equal to a 29% decrease. This decrease can be explained by the decrease in carbon dioxide content in syngas composition with increasing temperature.

### 4.3. Machine Learning Study

In order to develop machine learning algorithms, a total of 100 trials are designed with ten different levels of input variables. Specifically, the input variables selected are the steam-to-PVC waste ratio (SPR) and gasification temperature (T), with ranges of 1–3 for SPR and 1000–1300 K for T divided into ten levels. Of these random 100 trials, 90 are utilized to train the machine learning algorithms (trials number 1 to 90) while the remaining 10 are used to assess the accuracy of the developed algorithms (trials number 91 to 100). The training trials are presented in Table 2. Cold gas efficiency (CGE) and normalized carbon dioxide emissions (Emission) are the outputs to be predicted by machine learning algorithms and they are also presented in Table 2. To develop the regression algorithms, the regression analysis in the Minitab software is employed.

Firstly, the linear regression models are developed to predict the *CGE* and emission based on the linear terms. The following equations are presented for the linear algorithms:

(20) CGE=95.361−8.2601SPR−0.004294T

(21) Emission=288.09+46.603SPR−0.18766T

**Figure 6:**
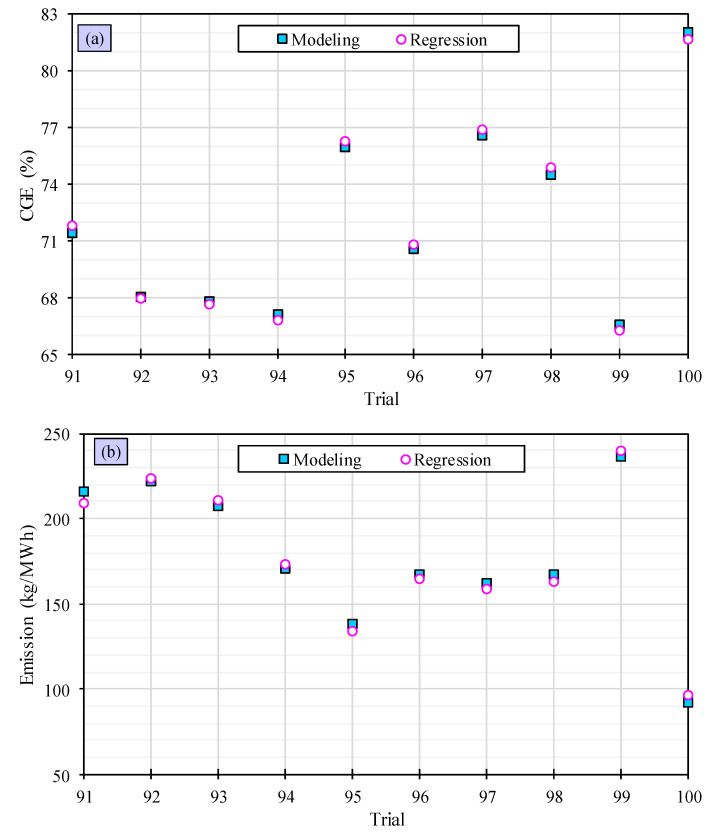
Evaluation of accuracy of linear machine learning algorithms for predicting the CGE (**a**) and the emission (**b**).

The precision of these algorithms is assessed and verified by contrasting them with the remaining 10 trials. The outcomes are shown in Figure 6. It is evident that the linear regression models are proficient at forecasting the *CGE* and emission with a high degree of accuracy, and their outcomes are closely aligned, indicating the favorable performance of the linear machine learning algorithms.

The quadratic regression models for predicting the CGE and emission are obtained as follows:

(22) $$CGE=98.55-11.8516SP-0.00424T+0.9011SPR^{2}-0.000000T^{2}$$

(23) $$Emission=489.9+100.90SPR-0.6259T-13.588SPR^{2}+0.000190T^{2}$$

**Figure 7:**
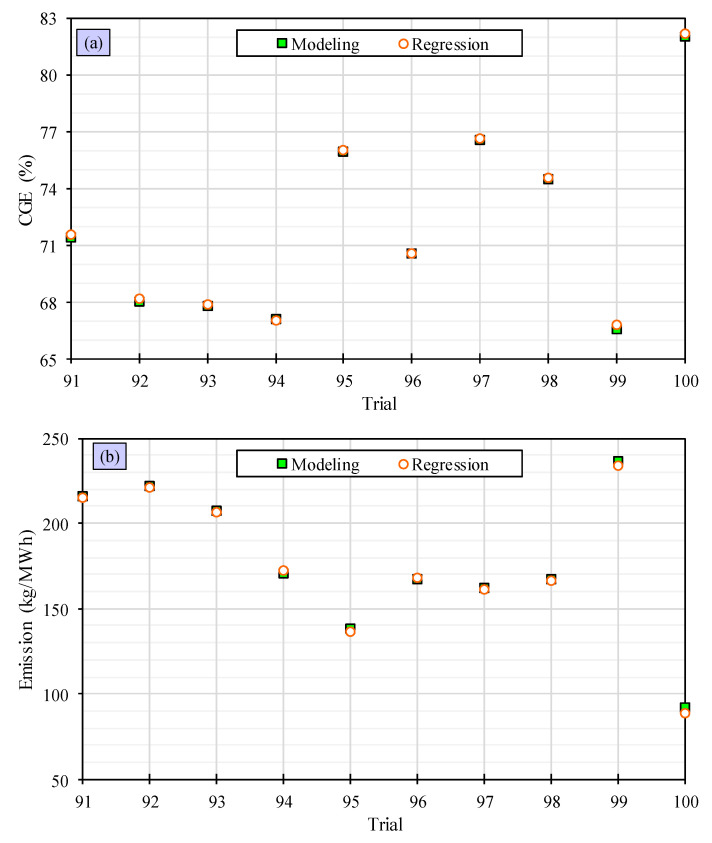
Evaluation of accuracy of quadratic machine learning algorithms for predicting the CGE (**a**) and the emission (**b**).

To ensure the accuracy of the quadratic algorithms, they are compared with the remaining 10 trials and the results are presented in Figure 7. The quadratic regression models were demonstrated to be highly accurate in forecasting the CGE and emission. Their outcomes are closely aligned, indicating their desirable performance. The observations confirm that the quadratic algorithms are more accurate than the linear ones and provide better predictions for the CGE and emission. This demonstrates the proficiency of the quadratic machine learning algorithms in this context.

The quadratic regression models considering the interaction term for predicting the *CGE* and emission are obtained as follows:

(24) $$CGE=100.528-13.1701SP-0.005438T+0.88437SPR^{2}-0.000001T^{2}+0.001202SPR\times T$$

(25) $$Emission=461.2+119.99SPR-0.6086T-13.346SPR^{2}+0.000198T^{2}-0.01740SPR\times T$$

**Figure 8:**
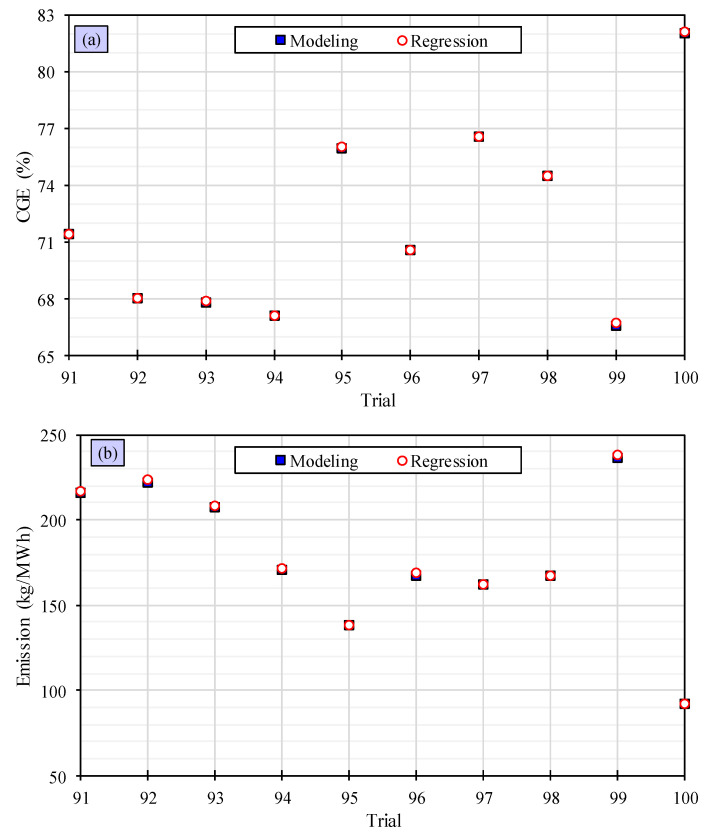
Evaluation of accuracy of quadratic machine learning algorithms by considering interaction term for predicting the CGE (**a**) and the emission (**b**).

In order to ensure the precision of the quadratic algorithms with the interaction term, the responses are taken into account and compared with the remaining 10 trials. The results, presented in Figure 8, show that the quadratic regression models with interaction term are highly accurate in predicting *CGE* and emissions. These models perform well, demonstrating their proficiency in this context. The observations indicate that quadratic algorithms with interaction terms are more precise than linear and quadratic ones, and they offer better predictions for *CGE* and emissions. Overall, this confirms the effectiveness of quadratic machine learning algorithms with interaction terms.

**Figure 9:**
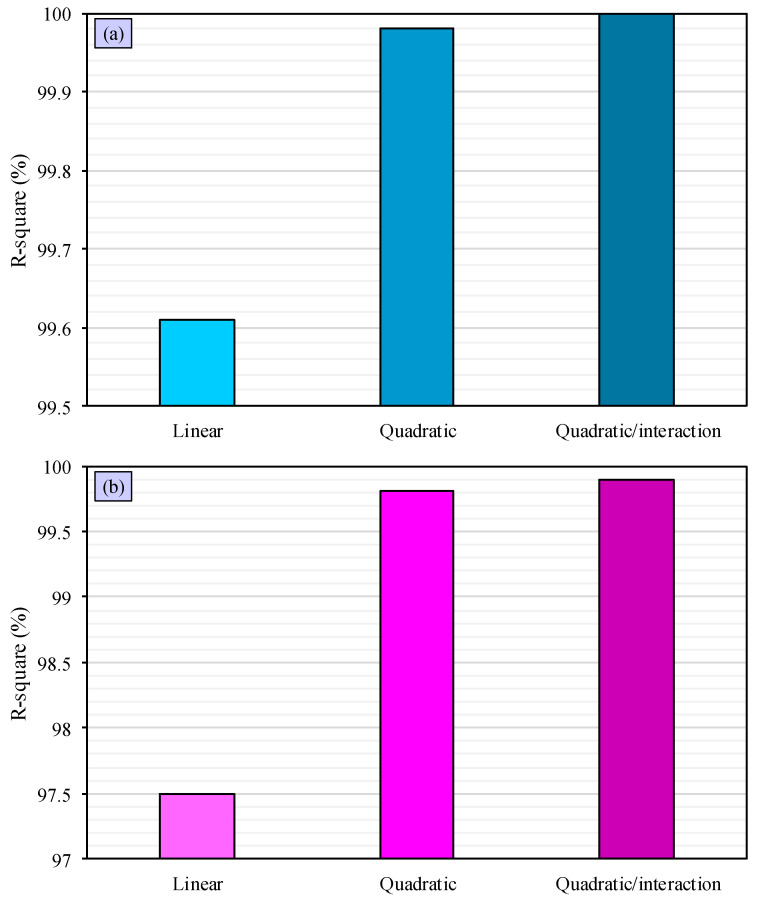
Comparison of different machine learning algorithms for CGE (**a**) and emission (**b**) with respect to R-square values.

In order to compare machine learning algorithms, their R-square values are measured and compared. The results are depicted in Figure 9, which demonstrates that the linear algorithm of CGE has an R-square value of 99.61%, indicating that it performs well at predicting the CGE. However, the quadratic algorithm performs even better, with an R-square value of 99.98%. The quadratic algorithm with interaction term has the best performance, predicting the CGE with an R-square of 100%. These findings suggest that the quadratic algorithm with interaction term is superior and has a greater predictive accuracy. Figure 9 illustrates the outcomes for emission algorithms, revealing that the linear emission algorithm has a high R-square value of 97.49%, indicating its strong predictive ability. However, the quadratic algorithm outperforms it, with an R-square value of 99.81%, while the quadratic algorithm with interaction term performs the best, with a perfect R-square value of 99.90%. These results indicate that the quadratic algorithm with interaction term is superior and has a higher predictive accuracy.

Predicted R-square estimates how well the model will perform on new data. Therefore, this value is measured for different regression models. The linear algorithm for CGE has a predicted R-square of 99.58%, while these values are 99.98% and 100% for predicted R-square of quadratic algorithm and quadratic algorithm with interaction term for CGE. These values for linear, quadratic, and quadratic with interaction algorithms for emission are 97.28%, 99.78%, and 99.87%, respectively. The significant high values of predicted R-square for all models show that all of regression models will perform well on new data.

Machine learning is revolutionizing the way we approach problem-solving in various fields, including PVC gasification. The future direction of machine learning application in the field of PVC gasification is promising, as it can help optimize the process and improve efficiency. With machine learning algorithms, it is possible to predict the behavior and performance of different PVC gasification performance indicators with respect to various process parameters, such as temperature, pressure, and feedstock composition. These predictions can be used to optimize the process and reduce the amount of energy and resources required. Another potential application of machine learning in PVC gasification is in the identification and classification of different types of waste plastics. By using machine learning algorithms, it is possible to identify the best types of plastics for gasification, which can help reduce waste and improve the efficiency of the process.

## 5. Conclusions

The purpose of this study was to assess the effectiveness of regression models as machine learning algorithms in predicting the performance of PVC waste gasification. The results indicated that the linear cold gas efficiency and emission algorithms had a good predictive ability with high R-square values of 99.61% and 97.49%, respectively. However, the quadratic algorithms performed even better, with R-square values of 99.98% and 99.81%, while the quadratic algorithms with interaction terms were the most superior, with perfect R-square values of 100% and 99.9%. Thus, it can be concluded that the quadratic algorithms with interaction terms are the most accurate and superior in terms of predictive accuracy. The outcomes of the study highlight the importance of utilizing regression models as machine learning algorithms in the field of PVC waste gasification. The future direction of machine learning application in the field of PVC gasification is promising, as it has the potential to improve the efficiency and sustainability of the process. By using regression machine learning algorithms, especially in combination with other techniques, it is possible to optimize the process and reduce the amount of energy and resources required, which can help reduce costs and improve the environmental impact of PVC gasification.

## Figures and Tables

**Table 1:** Chemical properties of PVC waste considered as feedstock.

Plastic	Proximate Analysis (wt%)				Ultimate Analysis (wt%)				Ref.
	Fixed Carbon	Volatiles	Moisture	Ash	C	O	H	N	
PVC	1.55	98.45	-	-	46.76	47.41	5.60	0.02	[49]

**Table 2:** Training trials considered for developing machine learning algorithms.

Run	SPR	T (K)	CGE (%)	Emission (kg/MWh)
1	2.778	1267	67.27	176.5
2	2.333	1033	71.35	208.2
3	1.222	1100	80.75	134.3
4	3.000	1100	66.31	213.8
5	1.667	1267	75.83	133.5
6	1.222	1000	81.25	156.4
7	1.444	1033	78.95	164.4
8	2.333	1100	71.10	192.8
9	2.556	1167	69.19	187.0
10	2.556	1300	68.71	164.5
…	…	…	…	…
81	3.000	1033	66.52	228.7
82	2.111	1233	72.31	158.9
83	2.778	1133	67.73	200.7
84	2.778	1000	68.16	230.6
85	1.444	1067	78.79	156.6
86	1.889	1067	74.82	181.5
87	1.889	1000	75.10	197.9
88	1.889	1033	74.96	189.5
89	2.556	1033	69.66	215.9
90	2.111	1000	73.24	207.8

## Data Availability

The data presented in this study are available on request from the corresponding author.
